# Decentralized digital twins of complex dynamical systems

**DOI:** 10.1038/s41598-023-47078-9

**Published:** 2023-11-16

**Authors:** Omer San, Suraj Pawar, Adil Rasheed

**Affiliations:** 1 School of Mechanical and Aerospace Engineering, Oklahoma State University, Stillwater, OK 74078 USA; 2 Department of Mechanical, Aerospace and Biomedical Engineering, University of Tennessee, Knoxville, TN 37996 USA; 3 Department of Engineering Cybernetics, Norwegian University of Science and Technology, 7465 Trondheim, Norway; 4 Department of Mathematics and Cybernetics, SINTEF Digital, 7034 Trondheim, Norway

**Keywords:** Applied mathematics, Computational science

## Abstract

In this article, we introduce a decentralized digital twin (DDT) modeling framework and its potential applications in computational science and engineering. The DDT methodology is based on the idea of federated learning, a subfield of machine learning that promotes knowledge exchange without disclosing actual data. Clients can learn an aggregated model cooperatively using this method while maintaining complete client-specific training data. We use a variety of dynamical systems, which are frequently used as prototypes for simulating complex transport processes in spatiotemporal systems, to show the viability of the DDT framework. Our findings suggest that constructing highly accurate decentralized digital twins in complex nonlinear spatiotemporal systems may be made possible by federated machine learning.

## Introduction

A recent multifaceted trend in science, engineering, and technology, both in academia and industry, is the digital transformation^1, 2^. The transformation has caught pace owing to cost-effective sensors, machine learning and artificial intelligence breakthroughs, improved computational infrastructure, and readily available course materials. However, this growth has resulted in several major challenges. First, the data exhibit a high correlation in many systems, which implies that more data does not necessarily translate into more information. Second, it is computationally demanding to train an end-to-end data-driven machine learning model that can be trustworthily used in future predictions. Third, there is a growing interest in the legal and regulatory landscape relevant to data sharing, security, privacy, and intellectual property rights (IPRs).

An outcome of rapid digitalization is the emergence of technologies such as digital twins. *A digital twin can be defined as an evolving computational model of an asset or process*^3–5^. The significance of digital privacy, primarily focused on safeguarding individual data privacy during statistical analysis, becomes increasingly important alongside the development of digital twin technology, which is primarily concerned with creating virtual replicas of physical systems for analysis and optimization^6–17^. Digital twins interact with physical assets in both ways: by controlling real assets and predicting their future states, and by calibrating models using data from the physical asset. The digital twin concept has gained a multibillion dollar market capitalization value for years to come, since it involves stakeholders from asset creation through decommissioning stages^18^. Numerous disruptive concepts and start-ups have been continuously evolving to bring more value and impact to society. According to a recent report by Research Dive^19^, the global digital twin market is predicted to grow at a compound annual growth rate of 40%, thereby garnering $125 billion by 2030. In particular, the availability of highly modular open-source libraries such as TensorFlow, PyTorch, and Theano, and the flexibility of cross-platforms such as Unity and Unreal Engine are lowering the barriers for many different potential use cases from precision agriculture^20^ to precision medicine^21^ and beyond. This open-source ecosystem has allowed scientists and engineers to expose themselves faster to the state-of-the-art digital twin technologies with cross-functionalities.

One of the key enablers of digital twins is modeling, which can either be first-principle physics-based modeling (PBM) or data-driven modeling (DDM) each with weaknesses that limits their usage in a digital twin context. To address the weaknesses, more recently, hybrid analysis and modeling (HAM)^4^ which is a fusion between PBM and DDM is rapidly evolving (see, e.g., see also^22–31^). Of the three modeling approaches, DDM and HAM thrive on the availability of a large amount of high quality data. The data requirement in the context of digital twins poses several challenges, especially as one moves towards creating digital twins of increasingly complex systems. We enumerate the challenges that are most relevant in the context of the current work:

- Any reasonably complex asset of a physical system or a process consists of multiple sub-components operated and maintained by different players who might have competing interests making data sharing difficult.
- Even when the IPRs involving data are sufficiently resolved, the shared big data characterized by 5Vs (large volume, velocity, veracity, variety, value) from different components/vendors can become overwhelmingly large that might require an exorbitant amount of compute power in one place to generate knowledge.
- Furthermore, the analysis of a large variety of big data in a centralized approach requires a wide array of expertise in one place, which will be difficult to achieve, resulting in sub-par analysis.
- Lastly, the limited bandwidth of data transmission in a centralized data analysis approach limits the amount of shared data.

To that end, the concept of federated learning can be utilized. Federated learning facilitates collaborative model training on decentralized devices (clients) housing local data, allowing updates to the global model without centralizing raw data, thereby preserving data privacy across distributed entities. Rich applications for training a machine learning model across numerous dispersed edge devices are emerging from this idea, and they do so without transferring local data samples across servers^32–35^. Although such privacy preserving decentralized and collaborative machine learning applications become more of a main stream approach in advertising, financial and many other industries with personalized dominance, it is relatively uncharted in advancing functionalities and providing technical support to forge a cross-domain, cross-data and cross-enterprise digital twin ecosystem that needs to be compatible with the diversity of scales present in data. Federated machine learning algorithms have been successfully used for training machine learning models in broader applications ranging from service recommendation to wireless communication. However, the multifidelity, multiphysics and multiscale nature of the many engineering and transport systems poses additional challenges because the accurate representation of each fidelity does not guarantee an improved metrics at the system level, partially because of nonlinear interactions across scales and also due to errors which may propagate and accumulate^36^.

In this study, we aim to extend the prospects of this concept to complex dynamical systems in order to provide building blocks (from a modeling perspective) for next generation decentralized solvers. With this in mind, our chief motivation is to communicate to a wide variety of audiences on how federated machine learning can address many of the issues raised above. Consequently, this is more of a concept paper, and we omit providing rigorous derivations of the utilized algorithms. Consistently, several relatively simple (but representative) problems have been chosen to demonstrate the framework. The plain vanilla versions of learning models have been utilized without any particular efforts dedicated to rigorous hyperparameter tuning and model calibration. Rather, our focus is laser sharp on highlighting the exploitation of the benefits of federated machine learning in the context of digital twin.

## Federated learning in the context of digital twins

### Digital twins

**Figure 1:**
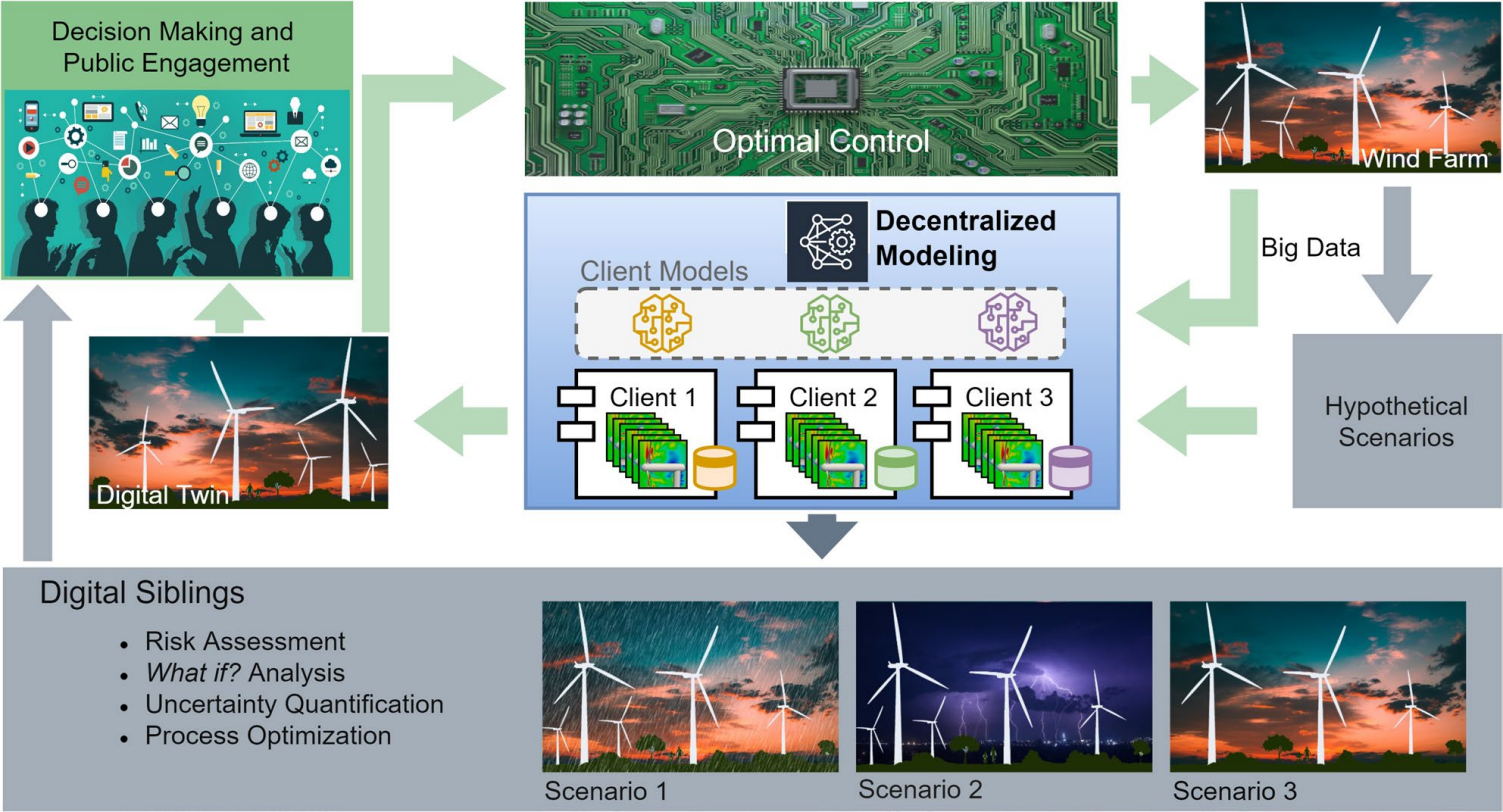
An overview of a decentralized digital twin concept for wind farm applications.

Digital twin, which is a virtual version of a physical asset that is made possible by data and simulators for improved decision-making and real-time prediction, optimization, monitoring, and control^5^, is a technology penetrating every domain. Figure 1 describes the concept of a digital twin utilizing a decentralized modeling for physical realism. On the top right side of the figure, we have the physical asset we want to represent by a digital twin. The physical asset is often equipped with a diverse class of sensors that give big data in real-time. This data has a very coarse spatio-temporal resolution and does not describe the future state of the asset. Therefore, to complement the measurement data, models are utilized to bring physical realism into the digital representation of the asset. Provided that the same information can be obtained from the digital twin as from the physical asset, we can utilize the digital twin for informed decision-making and optimal control of the asset. However, one might be interested in risk assessment, what-if? analysis, uncertainty quantification, and process optimization. These can be realized by running the digital twin in an offline setting for scenario analysis. The concept is then known as a digital sibling.

Additionally, the digital twin predictions can be archived during the asset’s lifetime and can then be used for designing the next generation of assets, in which case the concept is referred to as digital threads. Digital twins, based on their capability levels, can be categorized on a scale from 0 to 5 as standalone, descriptive, diagnostic, predictive, prescriptive, and autonomous. It is evident that modeling plays a vital role in enhancing the capability of digital twins. As pointed out in a recent survey^4^, any modeling approach should at least be generalizable, trustworthy, computationally efficient, accurate, and self-adapting.

Since a digital twin requires a real-time exchange of data between the physical asset and its digital representation, data privacy and security objectives become an increasingly important topic. Due to the participation of multiple stakeholders all the way from the development, through operation to the decommissioning phase of physical assets, the gathered data has to be secure both in terms of validity and privacy. Many digital twin systems and applications might involve different original equipment manufacturers, and they might want to keep their proprietary rights to secure their designs.

The predictive digital twins are now coined with precision sciences, such as precision agriculture^20^, precision medicine^21^ as well as precision meteorology^37^, in emphasizing their improved and localized forecasting capabilities. For example, we emphasize the penetration of data-driven modeling in precision meteorology, which often refers to accurate microscale weather forecasting that is of great importance in enabling solutions to water, food, energy, and climate challenges in the coming century^38^. Therefore, an increase in parameterization capabilities and characteristics along with enhanced in situ observations within the atmospheric boundary layer becomes a key concept to improve the accuracy of such microscale weather forecasting models^39^.

Our chief motivation in this study relies on the fact that a greater than ever penetration of sensors and smart devices (e.g., smart weather stations, smartphones, and smartwatches) has remained an unexplored technology in many spatiotemporal extended systems, and crowdsourcing data-driven modeling could be a key enabler toward their emerging digital twin applications. For instance, there will be more than 7 billion smartphones worldwide by 2025^40^. Compared to the few official meteorological stations distributed throughout the world, this number is large. While the use of data from a small number of edge devices may not result in reliable forecasts, processing data from several connected, smart devices that have sensors may revolutionize weather monitoring and forecasting. Numerous applications of smartphones toward citizen-centered monitoring, detection and warning systems have been discussed in a recent overview^41^ including examples from earthquake predictions to traffic, pavement and structural engineering projects. All of these case studies support the utilization of smartphones as cost-effective instruments for collecting real-time data. Moreover, as discussed in a recent report^42^, the Weather Company uses information from over 250,000 personal weather stations. Furthermore, Chapman et al.^43^ provided their insights on how using Netatmo weather sensors and the crowdsourcing data-driven modeling paradigm might advance the field of meteorology. The security and privacy risks of such linked and smart weather stations have also been highlighted as more focus switches to the internet of things (IoT) devices^44^. Additionally, we point readers to recent discussions on IoT uses for meteorological purposes and low-cost design of weather stations^45–48^. Furthermore, it is noteworthy that there is a contemporary inclination towards the utilization of federated large language models^49^. This trend is propelled by the fact that data generation is primarily driven by end users, and when coupled with advancements in wearable device technology^50^, federated models might exhibit a heightened effectiveness, particularly when harnessed in the context of generative models, for the purpose of adapting to screenless computing devices.

### Federated learning

**Figure 2:**
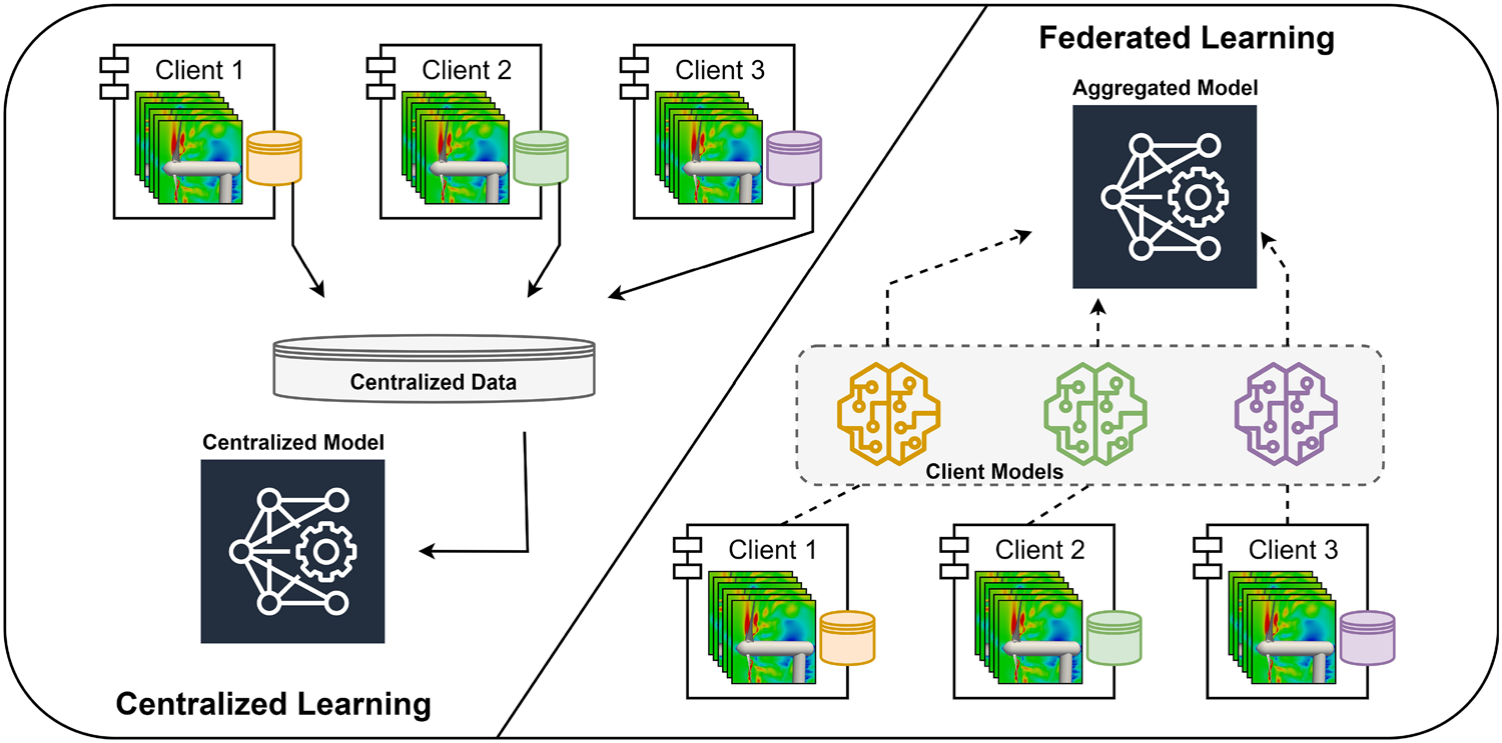
Overview and schematic illustrations of the centralized and federated machine learning approaches.

Federated machine learning is the collective training of machine learning models without the actual data being shared. We follow the pioneering work in federated learning^51^, which provides a federated averaging technique in which clients cooperatively train a shared model. The federated learning strategy is contrasted with the centralized approach in Fig. 2. Typically, the local dataset is moved from clients to a central server and the model is trained using data that is saved there when utilizing the centralized approach. However, the local dataset is never sent from clients to a server in federated learning. Instead, based on the local dataset, each client iteratively computes an update to the global model kept by the server, and only weights of the model are sent in each round. The federated averaging approach makes the assumption that a synchronous updating scheme is used in rounds of communications and that there is a fixed set of *K* clients with a fixed local dataset. The global state of the model, or the current model parameters, are sent by the central server to each client at the start of each communication round. Based on the global state and local dataset, each client computes the update to the global model, which is then communicated to a server. Following then, the server updates the model’s overall state using the local updates that it has received from each client, and the cycle repeats. The federated averaging algorithm’s objective function can be expressed as follows

1 $$\begin{aligned} f(w) = \sum _{k=1}^{K} \frac{n_k}{n}F_k(w), \end{aligned}$$

where

2 $$\begin{aligned} F_k(w)=\frac{1}{n_k} \sum _{i \in \mathscr {P}_k} f_i(w). \end{aligned}$$

Here, *w* denotes the parameters of the trainable model, $n_k$ denotes the cardinality of $\mathscr {P}_k$, $f_i(w)=l(x_i,y_i;w)$ denotes the loss of the prediction on sample $(x_i,y_i)$, and $\mathscr {P}_k$ denotes the data on the *k*th client. Any machine learning algorithm can be used with the aforementioned aggregation technique. We emphasize that the methodology we employ in our study^51, 52^ only weights edge devices proportionally according to the data they possess. These restrictions can be mitigated by more sophisticated methods^53–57^, but such methods are outside the purview of this paper.

In accordance with the provided pseudocode from prior research^52^, the primary server initializes the process by transmitting the global model state (i.e., model parameters) to all participating clients at the outset of each communication round. Subsequently, the central server accumulates these gradients and applies an update rule to the received data, producing the next iteration of model parameters through a weighted-average aggregation of local gradients. These updated parameters are then dispatched by the central server to the active clients. In essence, each client utilizes its local data to execute a single gradient descent step on the current model, after which the server computes a weighted average of the resulting models. This process is repeated for a specified number of gradient descent steps conducted by each client on their local dataset before transmitting their updates to the server. Consequently, varying federated algorithms can be formulated based on the number of training cycles executed by clients and the selection criteria for clients participating in each parameter update round.

## Demonstration cases

To demonstrate the value of the federated learning we chose the following frameworks and testbeds.

### Federated reduced order modeling framework

The one-dimensional Burgers equation, a common example of a nonlinear advection-diffusion problem, serves as our first test case for illustrating the DDT framework.

The viscous Burgers equation can be written as follows

3 $$\begin{aligned} \dfrac{\partial u}{\partial t} + u \dfrac{\partial u}{\partial x} = {\nu } \dfrac{\partial ^2 u}{\partial x^2}, \quad x \in [0,1], \end{aligned}$$

where $\varvec{u}(\varvec{x}) \in \mathbb {R}^{N_x}$ is the velocity field. We can write an analytical solution of the Burgers equation that satisfies Eq. 3 as follows

4 $$\begin{aligned} u(x,t) = \frac{\frac{x}{t+1}}{1 + \sqrt{\frac{t+1}{t_0}} \text {exp} \big ( \frac{x^2}{4\nu (t + 1)} \big )}, \end{aligned}$$

where $t_0=\text {exp}(1/8\nu )$. We parameterize the Burgers equation using the time *t* and viscosity $\nu$ as the parameter space, i.e., $\varvec{\mu }=(t,\nu )^T$. The first step of the reduced order modeling (ROM) is to generate the data. We generate the data for different values of parameters. Specifically, the parameter *t* is varied between 0 and 2, and the parameter $\nu$ lies between 0.001 to 0.01. The matrix $\varvec{A}$ $\in$ $\mathbb {R}^{N_x \times N}$, whose columns are the $\varvec{u}_n$ corresponding to $\varvec{\mu }_n$, is formed after the data is generated. The matrix is then subjected to singular value decomposition

5 $$\begin{aligned} \varvec{A} = \varvec{W}\varvec{\Sigma }\varvec{V}^T = \sum _{k=1}^N\sigma _k \varvec{w}_k \varvec{v}_k^T, \end{aligned}$$

where $\varvec{W}$ is an $N_x \times N$ matrix with orthonormal columns, $\varvec{w}_k$, $\varvec{V}$, which is an $N \times N$ square matrix with orthonormal columns, $\varvec{v}_k$, and $\varvec{\Sigma }$ is also an $N \times N$ matrix with non-negative diagonal entries, often known as singular values, arranged so that $\sigma _1 \ge \sigma _2 \ge \dots \ge \sigma _N \ge 0$. The proper orthogonal decomposition (POD) modes are represented by the vectors $\varvec{w}_k$, and the set of POD basis functions is represented by $\varvec{\Phi }=\{ \phi _k\}_{k=1}^{R}$^58^.

The following is then a representation of the approximate velocity field using POD modes

6 $$\begin{aligned} \varvec{u}(\varvec{x}, \varvec{\mu }) = \sum _{k=1}^{R} \alpha _k(\varvec{\mu }) \phi _k(\varvec{x}), \end{aligned}$$

where *R* is the number of modes that are retained in our model, and $\alpha _k{(\varvec{\mu })}$ denotes the *k*th modal coefficient.

The relative information content (RIC) can be used to determine how much energy POD modes maintain. We determine *R* from the RIC index given by

7 $$\begin{aligned} \text {RIC}(R) = \frac{\sum _{j=1}^{R} \sigma _j^2}{\sum _{j=1}^{N} \sigma _j^2} , \end{aligned}$$

where we set RIC(*R*) = 99.99%.

**Figure 3:**
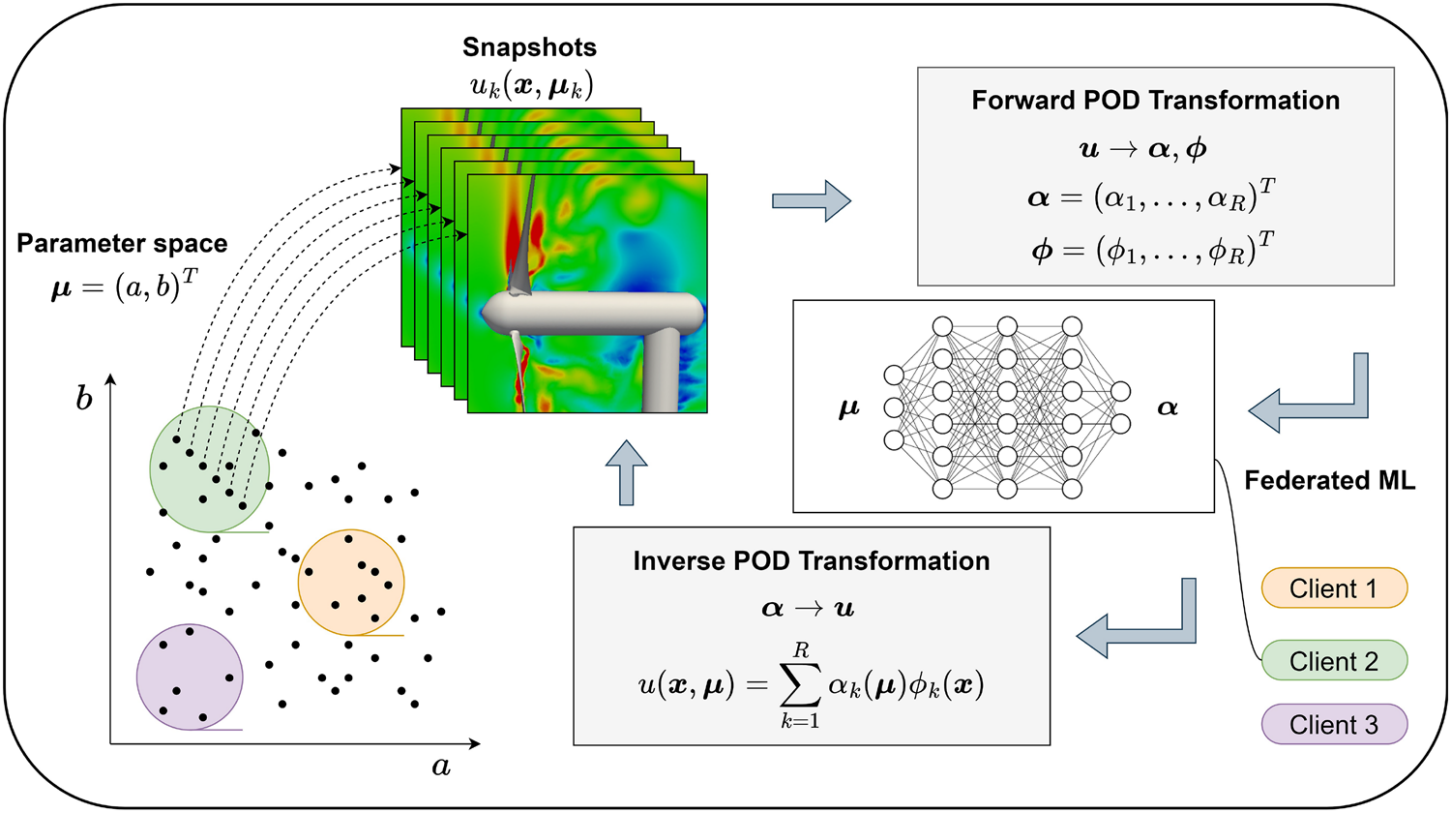
Federated reduced order modeling framework. This approach enables clients to collaboratively learn an aggregated model while keeping all the training data on each client. In other words, it encourages knowledge sharing without sharing the actual data. It deals with training a model across multiple decentralized edge devices (or servers) without exchanging local data samples between them.

We first project snapshot data to POD modes (i.e., forward POD transformation). The neural network is then trained to learn the relationship between the parameters of the Burgers equation to the POD modal coefficients. Once the neural network is trained, we can predict the POD modal coefficients for a new set of parameters (i.e., inverse POD transform), and the velocity field can be reconstructed using Eq. 6. In the case of the federated ROM framework, the low-dimensional data is assumed to be distributed across clients and the training is done using the federated averaging algorithm^51^. The complete federated ROM framework is illustrated in Fig. 3 where clients collaboratively learn an aggregated model while keeping all the training data on their respective client. The machine learning model is a feed-forward neural network with four hidden layers and forty neurons per hidden layer. We use the rectified linear unit (ReLU) activation function to introduce nonlinearity and the linear activation function is used at the output layer.

### Federated autoencoder framework

Our second test case is the decentralized autoencoder for the nonlinear dimensionality reduction of the NOAA OI SST V2 analysis dataset. Autoencoders are widely used in unsupervised machine learning tasks. Closely following^52^, we use an encoder network to map an input to a low-dimensional latent space, and a decoder network to translate latent variables back into the original inputs. This dataset has been created utilizing in-situ (ship and buoy) and satellite see surface temperature (SST) measurements along with sea-ice cover simulations of SSTs. The satellite data is corrected for biases^59, 60^ before the analysis is computed. This dataset is composed of the weekly average sea surface temperature on a $1^\circ$ latitude $\times$ $1^\circ$ longitude global grid ($180 \times 360$).

Despite seasonal fluctuations giving the SST dataset a strong periodic structure, complicated ocean dynamics produce interesting flow physics in this dataset. This dataset has been used in a variety of recent works, including multi-resolution dynamic mode decomposition^61^, flow reconstruction^62^, and geophysical emulation^63^.

More specifically, the autoencoder is utilized for nonlinear dimensionality reduction of the NOAA OI SST V2 dataset. The autoencoder is composed of the encoder function $\eta (w)$ which maps the high dimensional sea surface temperature field to low dimensional latent space and a decoder function $\xi (w)$ which maps the low dimensional latent space to the same sea surface temperature field. Both encoder and decoder are parameterized by the weights *w* and these parameters are learned through training. We can represent the reconstruction of the temperature field with the autoencoder as follows

8 $$\begin{aligned} \eta (w), \xi (w)&= \mathop {\mathrm {arg\,max}}\limits _{\eta (w), \xi (w)} ~\Vert \varvec{\theta } - (\eta (w) \circ \xi (w))\varvec{\theta } \Vert , \end{aligned}$$

9 $$\begin{aligned} \text {Encoder} {:}{=}\eta (w)&: \varvec{\theta } \in \mathbb {R}^N \rightarrow \varvec{z} \in \mathbb {R}^R, \end{aligned}$$

10 $$\begin{aligned} \text {Decoder} {:}{=}\xi (w)&: \varvec{z} \in \mathbb {R}^R \rightarrow \varvec{\theta } \in \mathbb {R}^N, \end{aligned}$$

where $\varvec{\theta }$ is the sea surface temperature field, $\varvec{z}$ represents the low dimensional latent space and *R* is the dimensionality of the latent space at the bottleneck layer of the autoencoder. Here, we train the autoencoder model using the data from October 1981 to July 2010 (1500 snapshots), and we compare the performance of the trained autoencoder model with the unseen data using the data collected after July 2010.

### Federated physics-guided machine learning framework

In our final demonstration case, we devise a framework integrating physics-guided machine learning (PGML) within a federated environment to construct a DDT model, which was then applied to lift coefficient data from various NACA standard airfoils for comprehensive training and testing across diverse conditions. As elucidated by Robinson et al.^64^ in their works, PGML operates as a HAM approach merging simplified theories to construct robust machine learning models, integrating this knowledge both during training and deployment phases. Consequently, the neural architecture, including hyperparameter optimization and the definition of nodes and layers, plays a pivotal role in PGML’s efficacy, with a trade-off observed: overly narrow networks exhibit limited expressiveness impacting predictions, while overly complex architectures, coupled with insufficient training data, can lead to overfitting and reduced generalizability, as indicated by an escalating validation loss after several training iterations.

The concept of injecting data into machine learning models revolves around the convergence of data sources related to a physical system, encompassing data from high-fidelity numerical simulations, experiments, or various data acquisition methods such as in-situ sensors, satellite observations, and field measurements, all contributing to the understanding of the system’s behavior as a unified reality. In this study, we extend our prior PGML model^65^ into a federated architecture. In the current context, the concept of inductive bias holds paramount importance for efficient DTT modeling. Inductive bias encompasses a set of assumptions regarding the underlying phenomenon or data structure, typically derived from prior knowledge and experience, enabling effective adaptation to novel data. In the context of federated computing, we advocate the incorporation of inductive bias into the internal layers of neural operators. Broadly, every assumption and belief concerning the data represents an instance of inductive bias, which mitigates prediction uncertainty in ML models and facilitates early resolution of learning and memory issues. It is worth noting that inductive bias proves particularly reliable for overparameterized systems, rendering our approach a viable strategy for decentralized digital twins.

**Figure 4:**
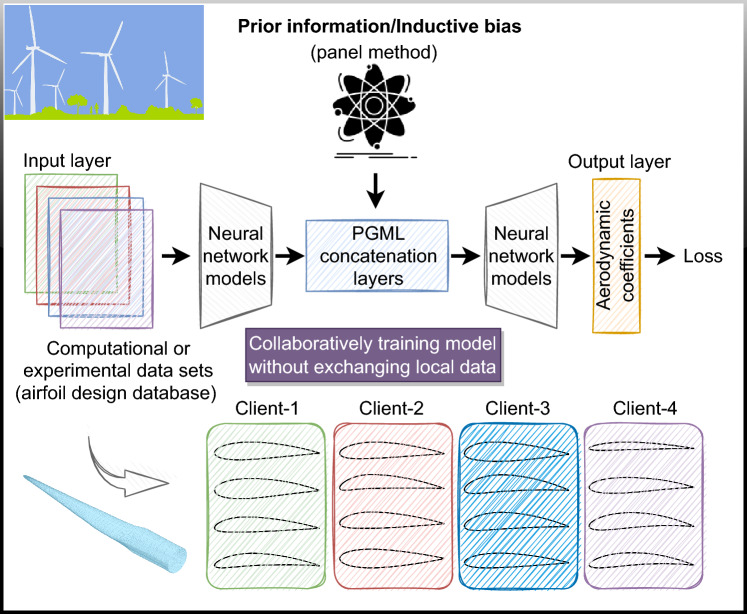
Illustration of the federated physics guided machine learning framework as a key enabler toward decentralized digital twins.

As shown in Fig. 4, each client possesses distinct airfoil geometries, and without sharing geometric specifics or relevant flow field data, our objective is to train a global model accommodating diverse local scenarios. Clients collaborate in learning an aggregated model while safeguarding the privacy of their individual training data. This decentralization of training data ensures data privacy, as raw data is not exchanged with the central aggregator. Clients utilize their own computational resources to perform local computations on their datasets, incorporating shared parameters based on the global model’s state. Consequently, the sole information transmitted to the server comprises the model updates generated by the clients using their local data. Upon receiving these updates, the parameter server adjusts its global model by updating the weights learned from each client’s local dataset through iterative iterations. In summary, our approach involves two critical phases: local updating with PGML network, where clients perform gradient descents to minimize the local loss function with respect to their data, and global aggregation, encompassing the collection of updated model parameters from multiple client devices, their consolidation, and the subsequent distribution of the aggregate parameters to clients for further training iterations. Utilizing the identical architecture as previously detailed in our earlier work^65^, we abstain from reiterating the specifics of the PGML network in this context. Instead, we present this example as an illustrative case showcasing the seamless extension of a specific PGML architecture into its federated counterpart while preserving its inherent characteristics and feasibility.

## Results

### Federated reduced order modeling framework: Burgers system

**Figure 5:**
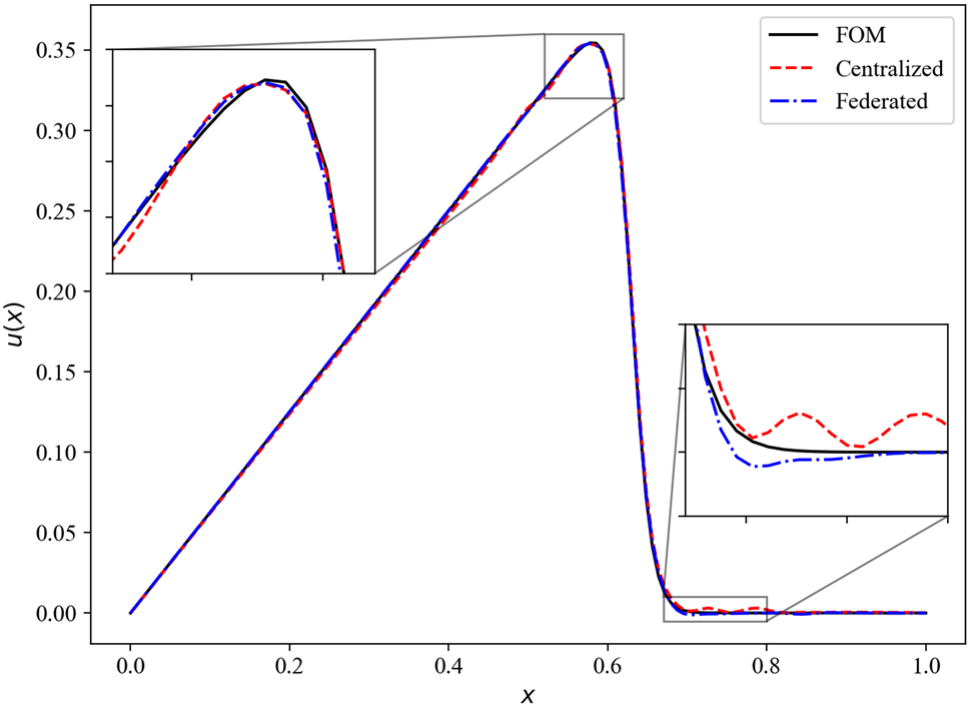
Validation loss during training of the centralized and federated neural network for the ROM framework of Burgers equation.

**Figure 6:**
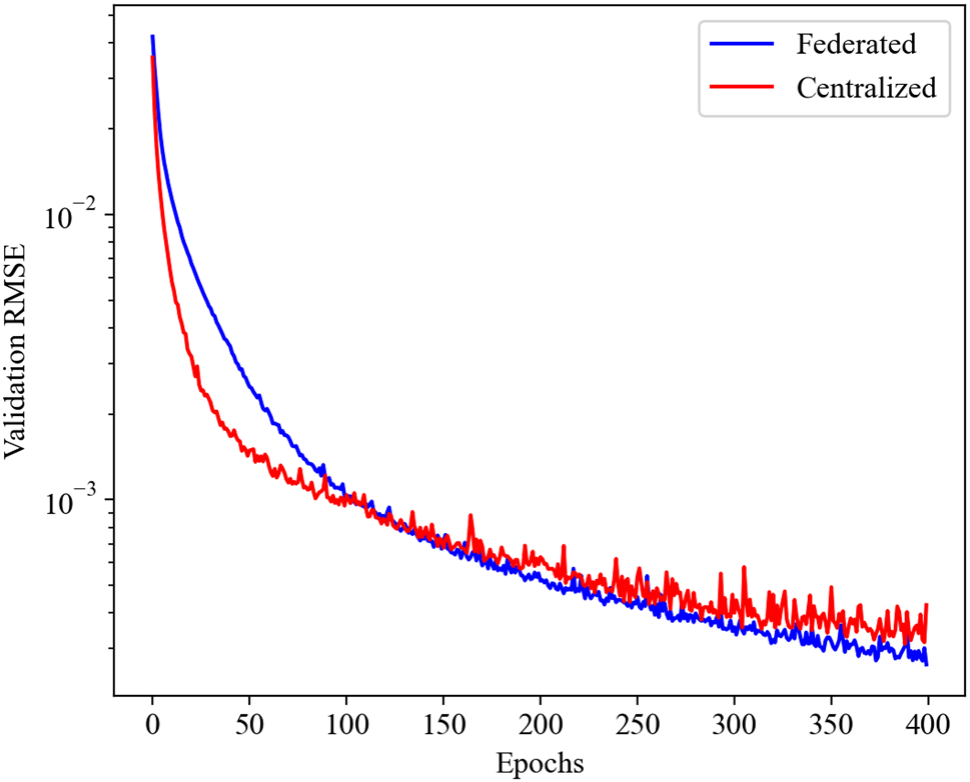
Reconstruction performance of the centralized and federated learning approaches for Burgers equation for $\varvec{\mu }=(0.02, 0.00475)^T$.

In our demonstrations, we employ $K=10$ clients for the federated learning, with each client model being trained for 5 local epoch with a batch size of 32. The centralized model is trained with a batch size of 320 to ensure fair comparison. The mean squared error loss for the validation dataset during the training of centralized and federated ROM frameworks is shown in Fig. 5. The validation loss for both centralized and federated learning follows a similar trajectory during the training indicating that there is no loss in federated learning. Therefore, federated learning can provide us with a deep learning model with a similar level of accuracy as centralized learning without the need to share the data. The performance of the parametric ROM framework for $\varvec{\mu }=(0.02, 0.00475)^T$ is shown in Fig. 6. Compared against the FOM data, which is the analytical (true) solution in this example, we see that both centralized and federated ROMs are highly accurate in reconstructing the velocity field for the Burgers equation.

**Figure 7:**
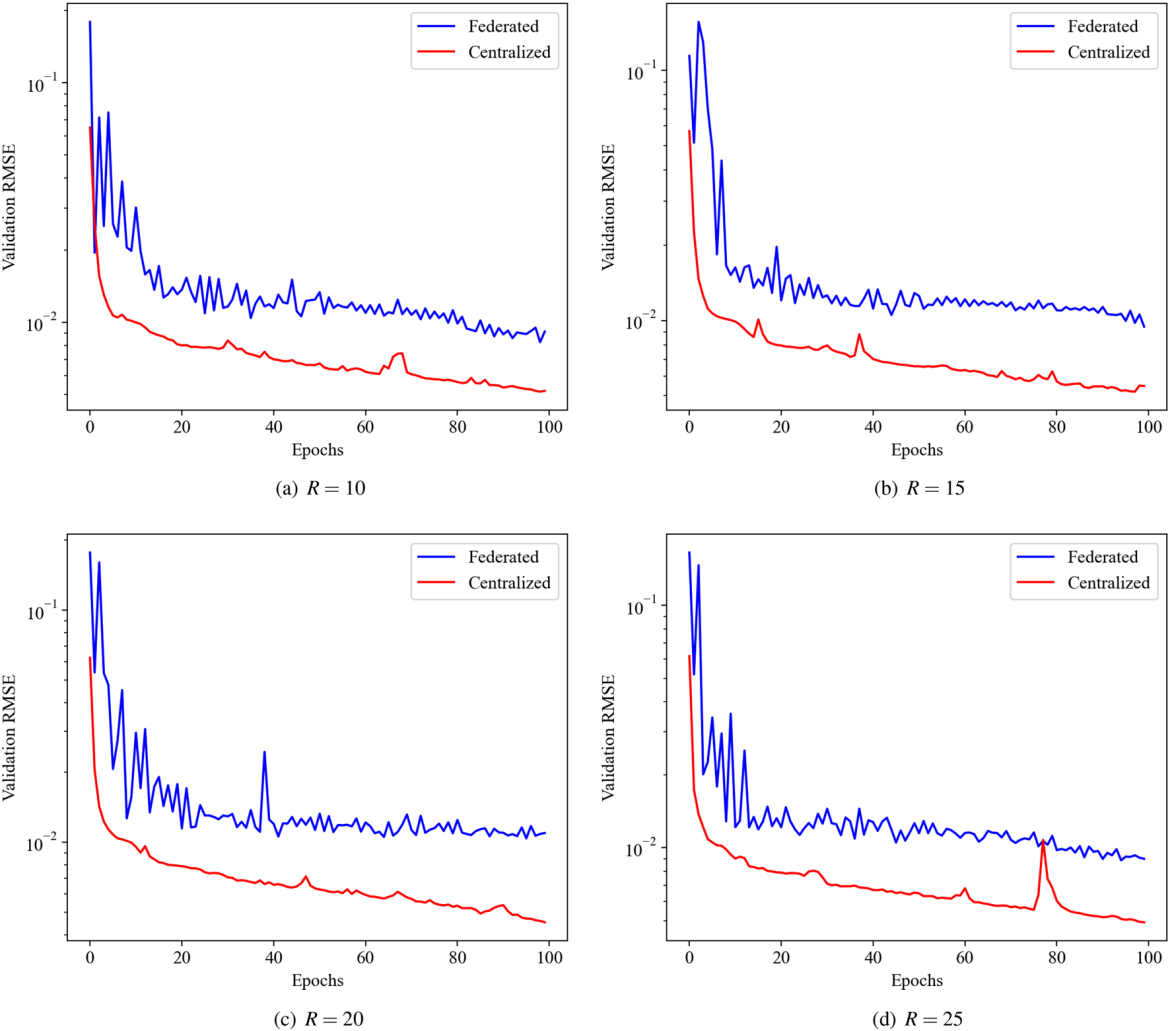
Validation loss during training of the centralized and federated neural network for the NOAA Optimum Interpolation Sea Surface Temperature Dataset.

**Figure 8:**
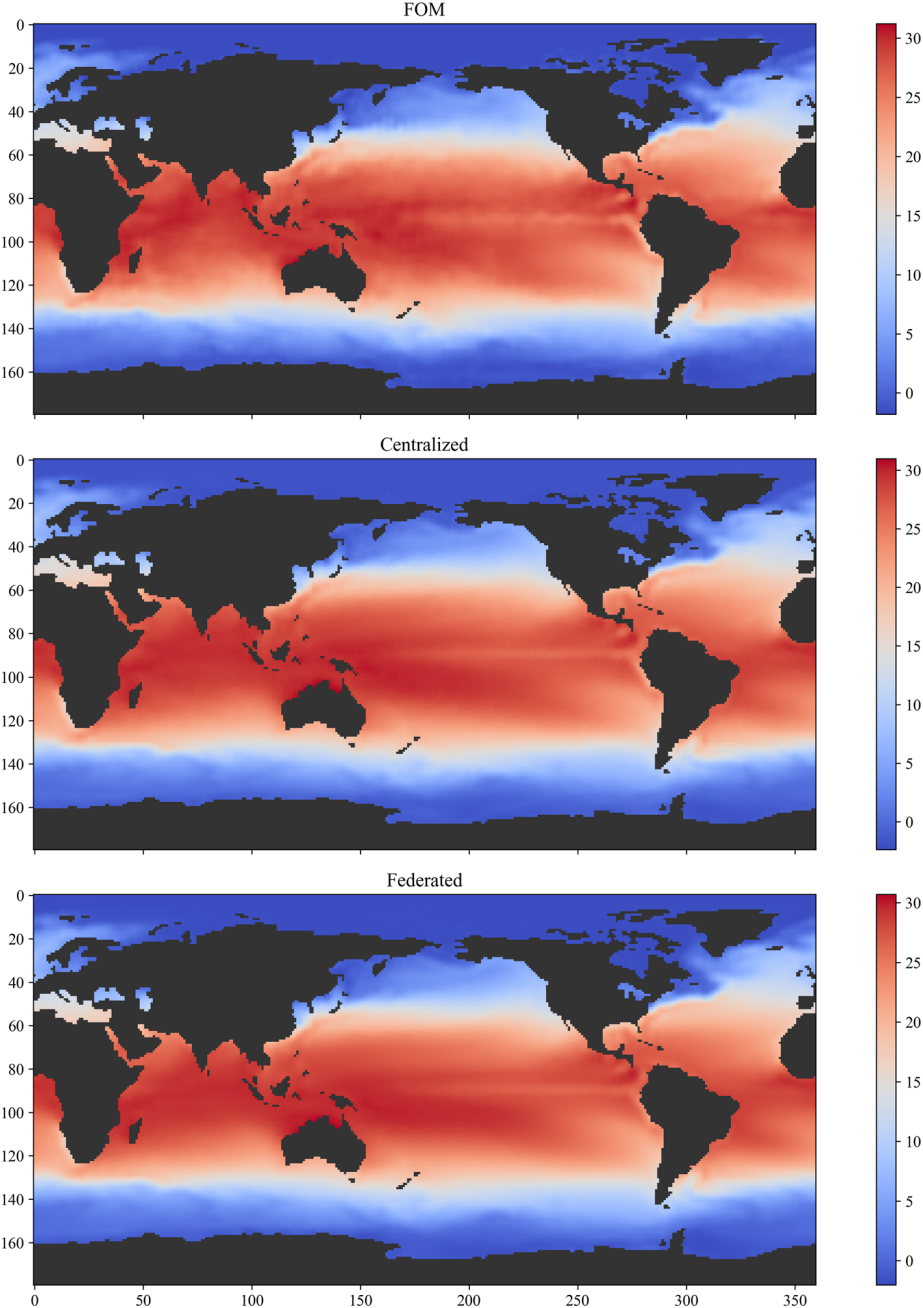
Weekly averaged temperature field in degrees Celsius for the fourth week of March 2018 along with reconstructed temperature field from the centralized and federated autoencoders.

### Federated autoencoder framework: NOAA optimum interpolation sea surface temperature dataset

First, we show the performance of centralized and decentralized or federated autoencoders for different dimensionality of the latent space at the bottleneck layer. The encoder is composed of three hidden layers with the number of neurons being $\{ 800, 400, 200\}$ across the first, the second, and the third hidden layer, respectively. The decoder has the same architecture in reverse order, i.e., three hidden layers with $\{ 200, 400, 800\}$ neurons. The number of neurons in the bottleneck layer is varied between $R=10$ to $R=25$ with an increment of 5. The encoder and decoder architecture remain the same for all the numerical experiments. In each case, we use $K=10$ clients, with each client model trained for 1 local epoch with a batch size of 16. To guarantee fair comparison, the centralized model is trained with a batch size of 160. Figure 7 depicts the validation loss during both centralized and federated training of the autoencoder. The validation loss for federated learning is slightly higher compared to the centralized learning from the first epoch. This can be attributed to the fact that in federated averaging the weights of the global model are initialized by simply averaging the weights of the client model. The simple averaging of weights might not be the most effective strategy especially when the amount of the data is small for each client. Despite this, the validation loss for both centralized and federated learning follows a similar trajectory with the loss reduced by almost one order of magnitude from the initial loss. The reconstruction of the weekly average temperature field for the fourth week of March 2018 is shown in Fig. 8 for both centralized and federated autoencoder along with the full order model, i.e., the analysis.

### Federated PGML framework: Aaerodynamic characterization

In this demonstration, the model was specifically trained, tested, and validated using NACA 4-digit and 5-digit series data pertaining to variations in the lift coefficient for diverse airfoil types. In line with the PGML model^65^, we extend its federated architecture to leverage pertinent physics-based characteristics from the panel method, enhancing the generalizability of data-driven models in a federated setting. For training purposes, numerical simulations are employed to generate data for a federated neural network involving $K = 10$ clients. The primary objective of this section is to compare federated and centralized approaches concerning prediction accuracy, both enriched with inductive bias and tested with distinct data sets from their training data. The training dataset encompasses lift coefficient data across various Reynolds numbers spanning from $1 \times 10^6$ to $4 \times 10^6$ and angles of attack ranging from $-20$ to $+20$, amounting to a total of 168 sets of two-dimensional airfoil geometries. This dataset covers NACA 4-digit, NACA210, NACA220, and NACA250 series airfoils, each with 201 points, featuring maximum thickness ratios ranging from 6% to 18% of the chord length. The testing dataset comprises NACA23012 and NACA23024 airfoil geometries, notably exceeding the maximum thickness ratios present in the training dataset and originating from a different NACA230 series not included in training.

Each client in the federated environment adopts a neural network architecture with four hidden layers, each containing 20 neurons. At the third hidden layer, latent variables and physical parameters, specifically the Reynolds number and angle of attack, are concatenated. By incorporating physical flow parameters, lift coefficient, and pressure drag coefficient predictions from the panel technique, we augment the latent variables at the third layer of the PGML model. The machine learning model consists of a feed-forward neural network with four hidden layers, each housing 20 neurons, and employs the ReLU activation function to introduce nonlinearity. To gauge predicted uncertainty, we employ an ensemble approach, combining predictions from multiple neural networks. These networks are trained with varying weight initializations and serve as an indicator of epistemic uncertainty. The ensemble of models is created by initializing each model’s weights and biases using the Glorot uniform initializer and employing various random seed numbers to ensure unique parameter sets for each model. The ensemble collectively provides an estimate of the expected lift coefficient model uncertainty.

**Figure 9:**
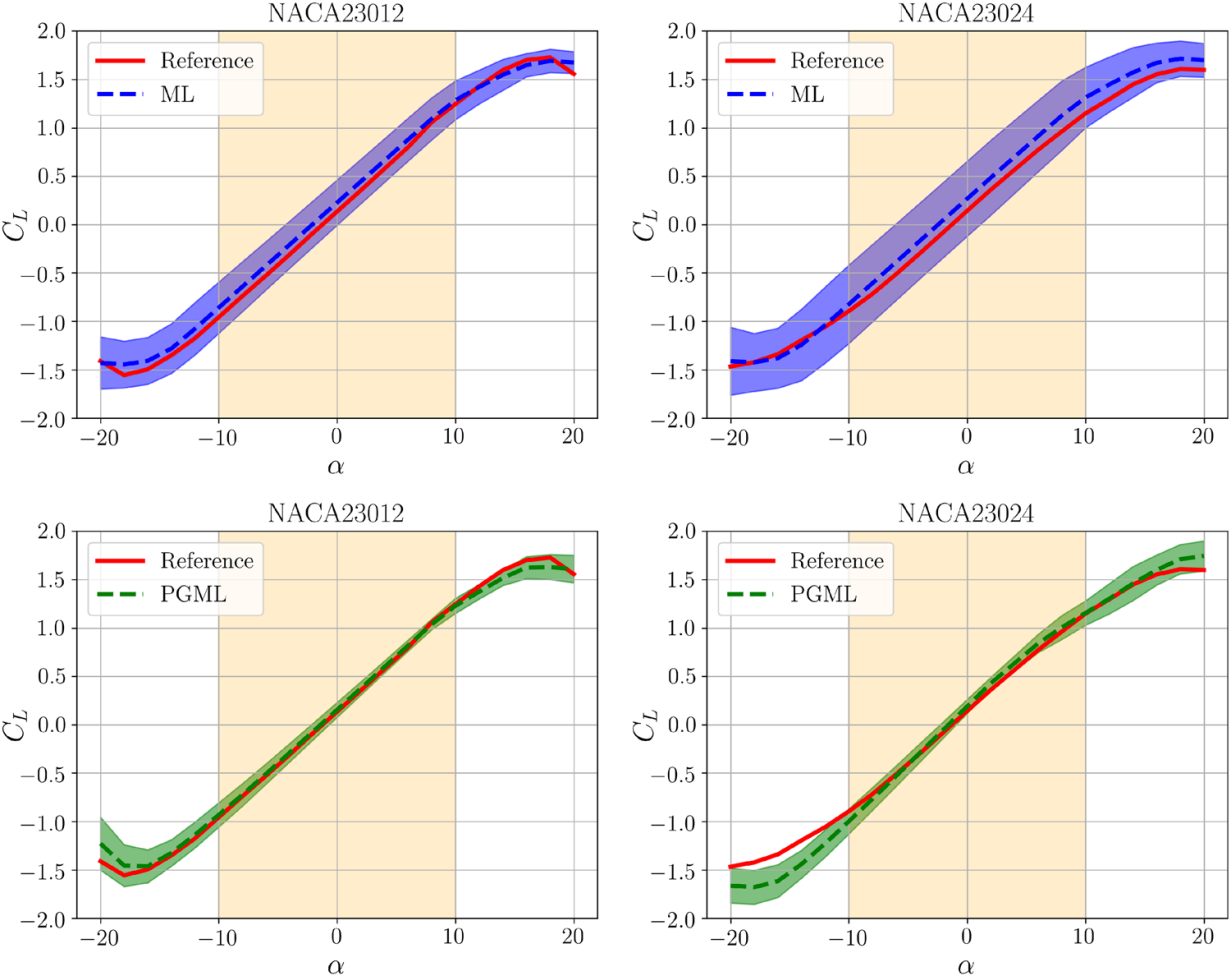
Uncertainly quantification of ML and PGML approaches using the centralized training.

Figure 9 presents the lift coefficient predictions for NACA23012 and NACA23024 airfoils to illustrate the difference between standard ML and PGML. The PGML model provides more precise lift coefficient predictions with reduced uncertainty compared to the ML model for both airfoil types, particularly within an angle of attack range of -10$^{\circ }$ to +10$^{\circ }$. Higher uncertainty is expected outside this angle of attack range due to the successful application of the Hess-Smith panel method in analyzing inviscid flow for smaller angles of attack. It’s worth noting that airfoil thickness in the training dataset does not exceed 18% of the chord length. The NACA23024 airfoil exhibits greater uncertainty in ML model predictions compared to the NACA23012 airfoil. The incorporation of physics-based information from the panel approach in the PGML model reduces this uncertainty. These findings highlight the advantages of enhancing neural network models with condensed theories and employing the PGML framework in physical systems.

**Figure 10:**
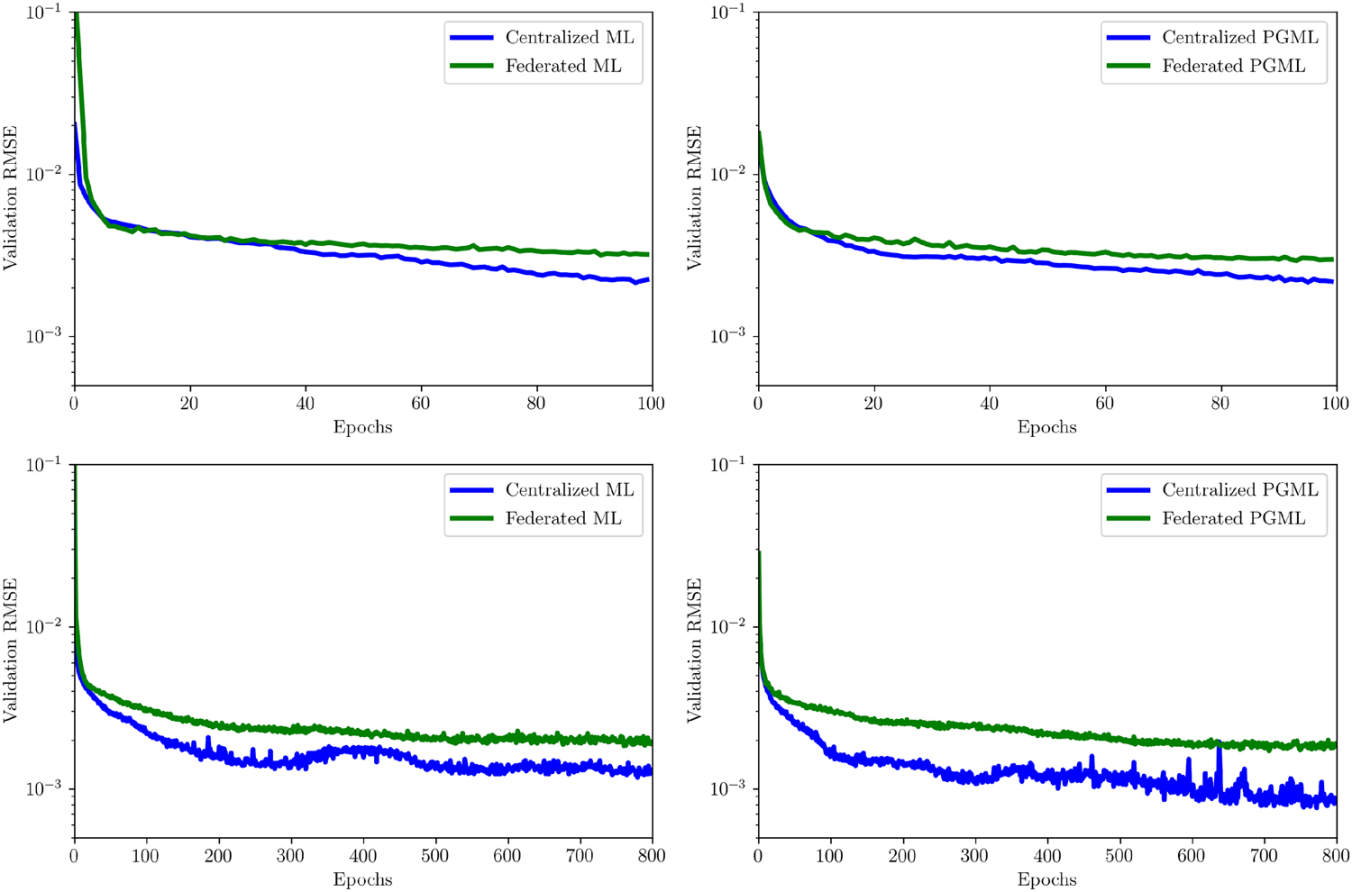
Validation loss of the proposed centralized and federated approaches. The first row shows the relaxation histories for the early truncation of 100 epochs, while the second row illustrates up to 800 epochs.

Figure 10 displays the reconstruction RMSE for centralized and federated modeling approaches, both for 100 and 800 training epochs. Divergent convergence trajectory characteristics are observed in the context of centralized and federated ML and PGML methodologies as the number of training epochs increases. While both approaches exhibit error values converging to similar levels, the PGML method demonstrates slightly lower errors, particularly in extended training epochs. It is noteworthy that federated learning plateaus at a higher error level, as indicated by the initial rapid reduction in loss within the first 100 epochs, followed by a more gradual decrease compared to centralized learning. This sharper convergence of the centralized approach results in a lower RMSE in loss, contrary to the trend depicted in Fig. 6. Consequently, our investigation suggests that instead of making a blanket assertion that federated learning is as effective as centralized learning, it is imperative to acknowledge the disparity in their convergence trajectories. This divergence may be attributed to a combination of various factors, including hyperparameters, batch sizes, data distribution imbalances, and the non-monotonic characteristics inherent to neural network-based approaches. Furthermore, the centralized model shows noticeable error fluctuations, absent in the federated model, due to the noise reduction effect in federated learning. This arises from the averaging of model parameter values contributed by participating clients.

**Figure 11:**
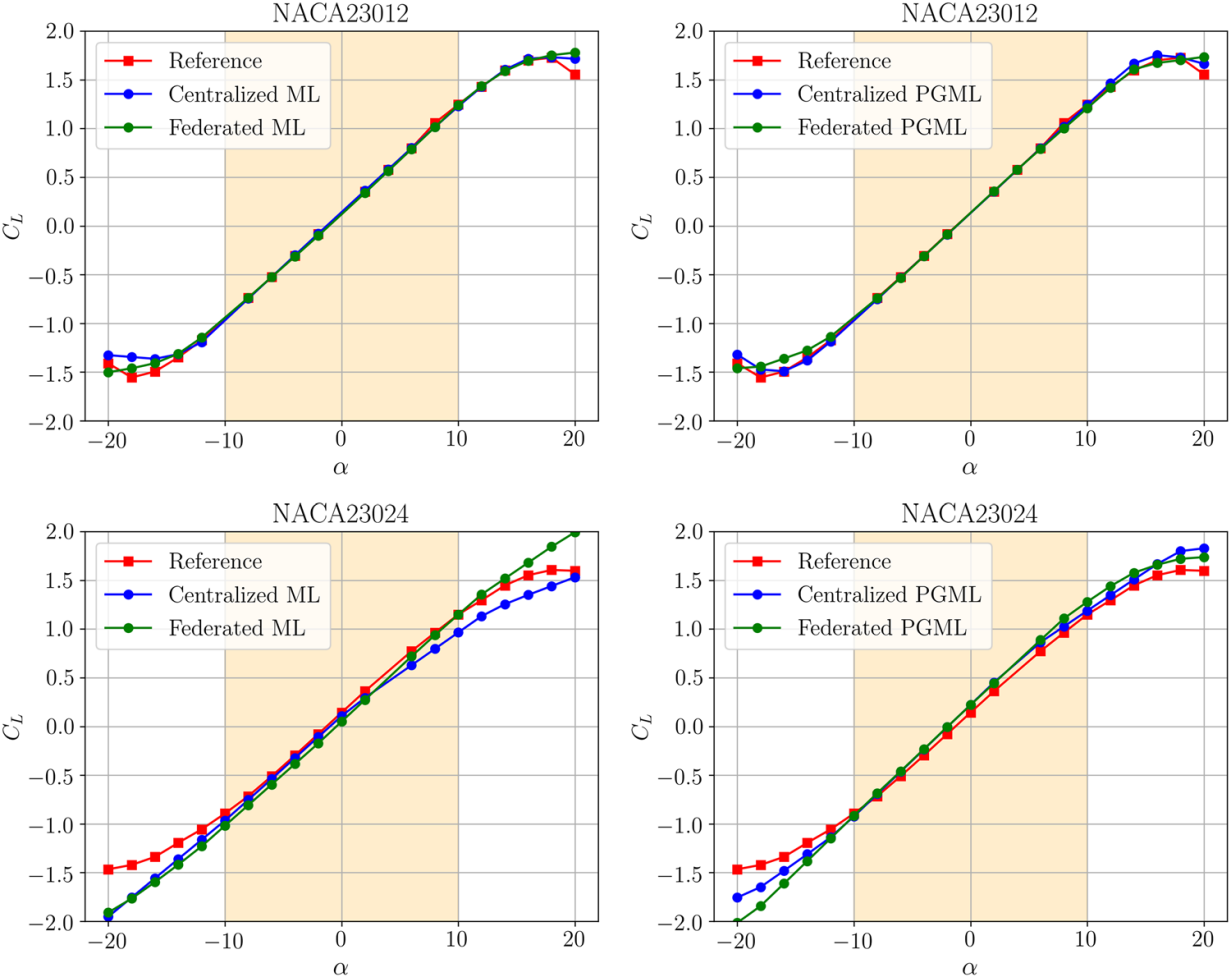
Predictive performance of the proposed centralized and federated models after 100 epochs.

**Figure 12:**
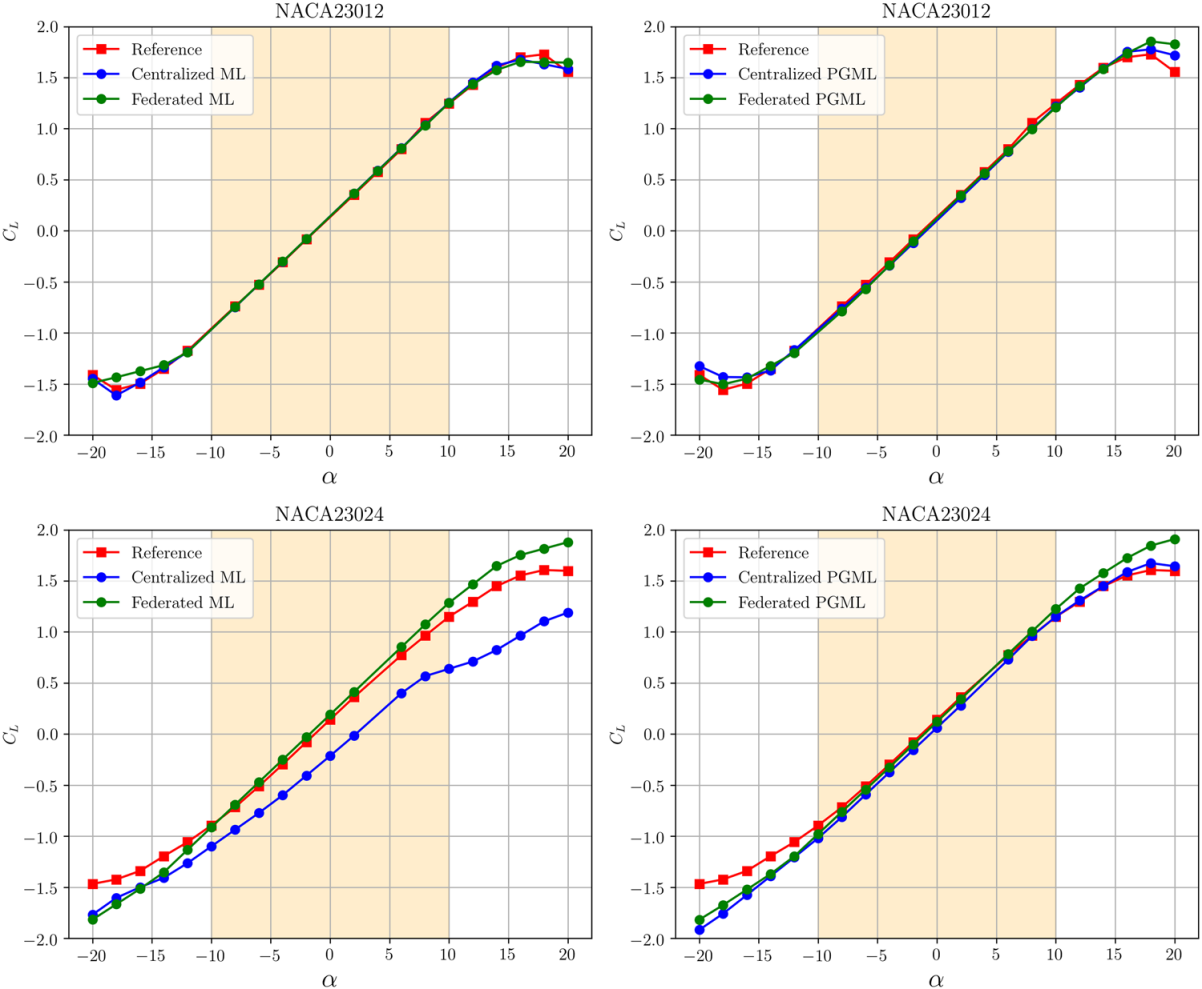
Predictive performance of the proposed centralized and federated models after 800 epochs.

Figure 11 investigates the models’ performance concerning the angle of attack for both NACA23012 and NACA23024 inputs, using both 100 and 800 training epochs. Predictions by all models are more accurate within the angle of attack range of -10$^{\circ }$ to +10$^{\circ }$, thanks to the superior accuracy of the panel method in this range. Models trained with NACA23012 data generally outperform those trained with NACA23024 data, which requires extrapolation, leading to deviations from the reference. Despite this, federated models demonstrate comparable or even superior performance, especially when tested with data outside the training range. The 800-epoch comparison in Fig. 11 highlights the stability of models trained with NACA23012 data throughout the entire angle of attack spectrum. However, with longer training epochs, a noticeable difference emerges between federated and centralized methods for models using NACA23024 data. Federated learning performs proportionally with centralized methods, underscoring the noise-canceling and filtering benefits of the federated environment, as shown in Fig. 12.

## Discussion

In this work, we explore the potential of federated machine learning in the context of a digital twin operated by multiple vendors to model complex spatio-temporal dynamical systems. In particular, we investigated federated learning for physics-guided neural networks and nonlinear dimensionality reduction of dynamical systems. Federated learning makes it possible to train a model jointly while maintaining the decentralized nature of the training data. Our numerical experiments reveal that a federated model does not achieve equivalent accuracy compared to a model trained on centralized data collected from all clients. Instead, our investigation emphasizes the importance of refraining from making definitive claims regarding the equivalence of federated learning and centralized learning. This divergence in convergence behaviors is attributable to a combination of factors, including hyperparameter configurations, batch sizes, data distribution disparities, and the non-monotonic traits inherent to neural network-based approaches. Nevertheless, it is essential to highlight that the federated learning process enables the updating of a global model without disclosing locally collected data from diverse sources. In summary, we provided insight for the following challenges:

- Since the data generated by individual vendors never leaves the local servers, a decentralized setting guarantees better security, and addresses key concerns related to IPRs. Furthermore, since each vendor can focus on individual sub-component of the asset, the data quality can be significantly enhanced.
- Since each data owner can conduct the training using the limited amount of data that they generate and own, they can work with significantly low compute power in a cost-effective way.
- Since the data are converted into knowledge which is generally a highly compressed representation, the knowledge transfer can take place over very limited bandwidth.

Although the primary focus of this study is federated learning in the context of spatiotemporal reconstruction of dynamical systems, the research findings may enhance broader modeling and simulation software capabilities for designing capable and secure predictive digital twins for cross-domain simulations. We discuss the prospects of such decentralized digital twin (DDT) modeling paradigm in various computational science and engineering applications. The DDT approach is built on a federated learning concept to collaboratively learn an aggregated model while keeping all the training data on each client. Our results indicate that federated machine learning might be a key enabler for designing highly accurate DDT applications involving complex nonlinear spatiotemporal systems.

In summary, the trained models contributed by the different stakeholder can be reversed engineered thereby compromising data security. To create new surrogate models that are compatible with heterogeneous computer environments, in our future studies, we intend to use decentralized learning methodologies in the context of precision science and create novel distributed physics-guided federated learning approaches. Also, we would like to investigate which federated learning approach is best for combining weights. The demonstration cases handled here are relatively simple, but that was completely intentional as it eases the communication and dissemination of the work to a larger audience. However, the next logical step should be to apply the approach to a more complex problem.

## Data Availability

The synthetic data that supports the findings of this study is available within the article. The weekly see surface temperature data can be found at the NOAA Optimum Interpolation (OI) SST V2 webpage: https://psl.noaa.gov/data/gridded/data.noaa.oisst.v2.html. The datasets used and/or analysed during the current study are also available from the corresponding author on reasonable request.
